# Beyond motor dysfunction: comorbidities in children with cerebral palsy: a retrospective observational study from a tertiary paediatric hospital in the UAE

**DOI:** 10.3389/fped.2026.1776355

**Published:** 2026-07-22

**Authors:** Lana Talo, Kerat Hanspal, Besher Alnukari, Hadi Helali, Sukanya Vrushhabhendra, Samar Almuntaser

**Affiliations:** 1 College of Medicine, Mohammed Bin Rashid University of Medicine and Health Sciences, Dubai Health, Dubai, United Arab Emirates; 2 Paediatric Neurology, Al Jalila Children’s Hospital, Dubai Health, Dubai, United Arab Emirates; 3 Paediatric Neurology, Mohammed Bin Rashid University of Medicine and Health Sciences, Dubai Health, Dubai, United Arab Emirates

**Keywords:** cerebral palsy, comorbidity, epilepsy, global developmental delay, pediatric neurology, rehabilitation, United Arab Emirates

## Abstract

**Background:**

Cerebral palsy (CP) is associated with comorbidities affecting motor, cognitive, and sensory function, which may be present at various levels in different patients, across regions, or within the same region. The comorbidity burden and the range of comorbidities in children with CP may vary according to disease severity, perinatal factors, and access to healthcare services.

**Objectives:**

To evaluate comorbidities, functional limitations, and rehabilitation use in children with CP in a tertiary hospital in the UAE and compare findings with international data.

**Methods:**

This retrospective observational study analysed 179 children with CP aged 1 month to 18 years, categorized into spastic quadriplegic, diplegic, hemiplegic, and unspecified subtypes. Data from electronic medical records were used to assess demographic characteristics, aetiologies, comorbidities, and rehabilitation engagement. Categorical variables were compared using chi-square tests. *P*-values were calculated for all comorbidities; no formal correction for multiple comparisons (e.g., Bonferroni or false discovery rate adjustment) was applied, and these analyses are considered exploratory and should be interpreted appropriately.

**Results:**

The cohort included 40.2% spastic quadriplegia, 34.6% spastic diplegia, 13.4% spastic hemiplegia, and 11.8% unspecified cases. Most children (58.1%) were born at term, with perinatal asphyxia (26.3%), hypoxic-ischaemic encephalopathy (14.5%), and neonatal jaundice (14.0%) being the most common aetiologies. Global developmental delay was the most prevalent comorbidity (78.6%), followed by drug-resistant epilepsy (63.7%). Visual impairments (43.6%), gait abnormalities (44.1%), and speech impairments (41.3%) were also common. A higher observed burden of comorbidities was noted in SQ compared with other subtypes, including feeding difficulties (43.1%) and musculoskeletal abnormalities (37.5%). Rehabilitation services were accessed by 64.8% of the cohort, primarily physiotherapy. Comparisons with international studies revealed similar trends in CP subtypes and comorbidities but highlighted disparities in aetiological patterns and access to rehabilitation.

**Conclusions:**

Children with CP in the UAE have a high comorbidity burden, particularly in severe subtypes. These findings support the importance of optimizing perinatal care and improving access to rehabilitation services, though prospective studies are needed to establish causal relationships

## Introduction

Cerebral palsy (CP) is a group of permanent, non-progressive disorders that affect movement and posture, resulting from early insults to the developing brain during prenatal, natal, or early postnatal periods (1, 2). Hypoxic events, congenital brain abnormalities, and infections during these phases have been associated with CP (3–6). With a prevalence of about 1.5–3 per 1,000 live births, CP is considered a leading cause of childhood debility worldwide, drastically impacting a child's function and growth (7, 8). Initially, CP was only characterized based on specific movement disorders, such as spasticity, dyskinesia, ataxia, or mixed/other, with spasticity being the most common and occurring in 80% of children with CP (9). However, beyond motor dysfunction, CP is often associated with a range of additional challenges, including disturbances in sensation, perception, cognition, communication, and behaviour (7, 10). This condition is associated with significant morbidity and mortality (3, 11).

Research in high-income regions such as the United States has identified common coexisting conditions, including intellectual disabilities, epilepsy, musculoskeletal abnormalities, and sensory impairments such as vision and hearing deficits (12). Studies from other parts of the world, such as Europe, reveal similar patterns, where epilepsy and orthopaedic complications like hip dysplasia and scoliosis are highly prevalent (13). However, data from the Middle East, and specifically, Dubai, remains scarce, creating a knowledge gap regarding the specific health challenges faced by children with CP in this region.

The prevalence and types of comorbidities among children with CP are known to vary based on socioeconomic, cultural, and healthcare access factors, underscoring the importance of region-specific data (14). In Dubai, research on CP comorbidities is limited, with few studies exploring the full range of complications, from neurological issues to systemic disorders, that these children experience (15).

The aetiology of CP varies widely between low- and middle-income countries (LMICs) and high-income countries (HICs). In LMICs, CP is primarily associated with preventable factors such as perinatal asphyxia, Hypoxic-Ischemic Encephalopathy (HIE), prenatal infections, neonatal hypoglycaemia, and hyperbilirubinemia. Meanwhile, HICs have noted a shift toward prematurity, genetic factors, and unidentified pathophysiological processes. These differences result in distinct CP subtypes, comorbidities, and functional limitations across regions. While CP aetiology and outcomes are well-documented in HICs, such as the U.S. and Europe, research in LMICs, particularly in the Middle East, remains sparse, with birth asphyxia and neonatal infections being the most common causes (3, 14, 16–18).

This study evaluates comorbidities in children with CP in Dubai and compares findings with international data to identify regional differences in epidemiology, presentation, and management, including the influence of healthcare access, environmental factors, and cultural practices.

## Materials & methods

### Study design and setting

This was a retrospective observational study conducted at Al Jalila Children's Specialty Hospital. The hospital serves both Emirati citizens and expatriate families. The study period spanned from January 16, 2019, to January 1, 2023. The study included all paediatric patients aged 1–18 years diagnosed with cerebral palsy (CP). The Institutional Review Board (IRB) of Mohammed Bin Rashid University of Medicine and Health Sciences approved the study (MBRU IRB-2024-86). Informed Consent from the patients was waived by the MBRU IRB, given that the study is a retrospective chart review, hence carries minimal to no risk, and can be carried out with the waiver, and because all the patients admitted to the hospital signed a consent on admission that their chart data will be used in future research.

### Participants and eligibility criteria

All patients aged 1 month to 18 years seen in outpatient clinics with a diagnosis of cerebral palsy during the study period were eligible. The cerebral palsy diagnosis, GMFCS classification, and subtype categorization were based on formal clinical assessments documented by paediatric neurologists. Exclusion criteria were: (1) age <1 month or >18 years; (2) incomplete birth-related data; (3) other classes of neurodegenerative disorders. These criteria were chosen to create a homogenous cohort and minimise confounding.

### Sample size (power calculation)

The prevalence of comorbidities accompanying cerebral palsy (CP) in the population was estimated to range from 10% to 85%. In view of these proportions, with a 95% confidence interval (CI) and a 10% absolute error margin, the minimum sample size was calculated to be approximately 49 participants. Our study commenced with a sample size of 227 participants, of which 179 remained after exclusions, meeting and exceeding the required sample size. This larger sample size improves the reliability of the findings and allows for more detailed analysis across CP subtypes.

### Data sources and variables

Electronic medical records were screened using Epic (locally referred to as “Salama”). Lists of clinic visits were filtered to identify infants with a clinical diagnosis of cerebral palsy. Patient charts were then reviewed to confirm eligibility. Data were abstracted into a password-protected spreadsheet. Data collection involved a detailed review of existing datasets and medical records available in the Salama database, with a focus on paediatric patients diagnosed with CP. The data extracted included demographic information, clinical characteristics, and comorbidities associated with CP. Key variables recorded were comorbidities such as epilepsy, intellectual disability, visual and hearing impairments, communication difficulties, and musculoskeletal disorders. To minimize misclassification bias, comorbidities were strictly extracted based on standardized operational definitions (Supplementary Table S1). A condition was only marked as present if confirmed by specialist assessment (e.g., paediatric neurology, orthopaedics, ophthalmology) or validated by objective testing (e.g., EEG, neuroimaging, or audiological testing), rather than relying solely on generalized chart notation.

The primary data sources were electronic medical records and standardized assessment forms used in clinical practice at the hospital. Outpatient clinic records were thoroughly reviewed to document comorbidities and demographic information, including patient age, gender, and clinical presentations. Neurological evaluations and comorbidity data were captured systematically, relying on assessments documented in the medical records without requiring direct patient contact. Existing neuroimaging data, when available, were also reviewed to support the classification of CP subtypes and to identify potential underlying brain abnormalities.

### Statistical analysis

Data were analysed using IBM SPSS Statistics version 28 (IBM Corp., Armonk, NY, USA). The baseline data were assessed for normality to determine the appropriate statistical measures. Continuous variables, including age in months, age at presentation, birth weight, and NICU days, were summarized using means and standard deviations (SD). Categorical variables were compared across CP subtypes using chi-square tests. Analyses were conducted using available data, and cases with missing values were excluded on a variable-specific basis. Missing data were assumed to be missing at random, primarily due to isolated documentation omissions in the electronic medical records. A two-sided *p*-value < 0.05 was considered statistically significant. Importantly, due to the small sample sizes within specific subgroups, the expected cell count assumptions for the chi-square test were not universally met. Furthermore, no formal correction for multiple comparisons (e.g., Bonferroni) was applied. As such, all inferential statistics and *p*-values reported in this study have been deliberately downgraded to be strictly exploratory and descriptive in nature. They should be interpreted merely as observational guides rather than definitive inferential evidence.

Data were stratified by type of CP into subgroups, including spastic diplegia (SD), spastic hemiplegia (SH), spastic quadriplegia (SQ), and unspecified (U). The proportion of children with CP presenting with each comorbidity was calculated and presented as percentages, allowing for comparisons across the different types of CP. Descriptive statistics were used to characterize the study population and summarize the prevalence of comorbidities. On the other hand, categorical variables were compared across CP subtypes using chi-square tests. Analyses were conducted using available data, and cases with missing values were excluded on a variable-specific basis. A two-sided *p*-value < 0.05 was considered statistically significant. No formal correction for multiple comparisons (e.g., Bonferroni or false discovery rate adjustment) was applied; therefore, the results should be interpreted as exploratory and with caution due to the increased risk of Type I error.

### Operational definitions of comorbidities

Comorbidities were extracted from the electronic medical record and input as present/absent depending on existing operational definitions to minimize misclassification bias (Supplementary Table S1). A comorbidity was considered present if documented as a diagnosis or recurrent presenting complaint by the relevant specialist (e.g., pediatric neurology, orthopedics, ophthalmology, audiology, physiotherapy) in clinician notes or the diagnosis list; objective testing and supporting documentation were utilized where available (e.g., EEG reports for epilepsy, ophthalmologic or audiological assessments for sensory impairments, and relevant imaging studies such as brain imaging and radiographs).

## Results

### Participants

**Figure 1:**
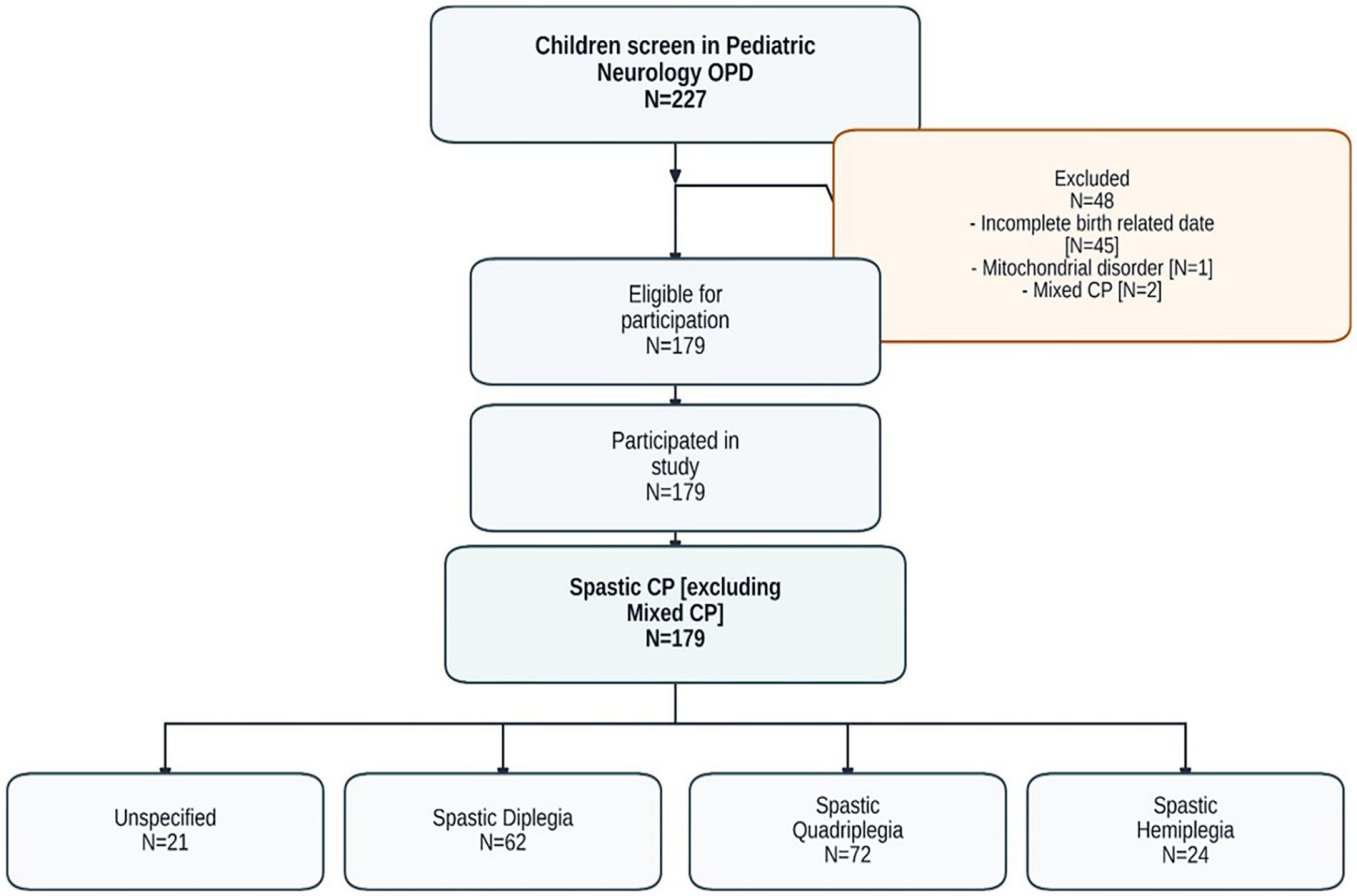
Flow chart of patients.

Out of 227 children screened, 179 met the inclusion criteria for the study (Figure 1). Of them, 158 (88.2%) had spastic CP, classified as spastic diplegia (SD, *n* = 62, 34.6%), spastic hemiplegia (SH, *n* = 24, 13.4%), and spastic quadriplegia (SQ, *n* = 72, 40.2%). Additionally, 21 children were classified as unclassifiable (11.7%). No child in this cohort had ataxic CP.

### Demographic characteristics

Children in this cohort had a mean age of 90.3 months (SD 56.6) (Table 1). Mean gestational age at birth was 36.7 weeks (SD 3.8); 104 patients (58.1%) were born at term, while 28 (15.6%) were very preterm, 11 (6.1%) moderately preterm, 15 (8.3%) late preterm, and 20 (11.1%) extremely preterm.

**Table 1:** Demographic characteristics of children with cerebral palsy according to subtype (*N* = 179).

Characteristic	Types of cerebral palsy							
	Spastic diplegia *n* = 62		Spastic hemiplegia *n* = 24		Spastic quadriplegia *n* = 72		Unspecified *n* = 21	
	Mean	Standard deviation	Mean	Standard deviation	Mean	Standard deviation	Mean	Standard deviation
Age (Months)	87.7	52.3	90.0	52.9	96.6	57.1	86.9	63.9
Age at Initial Presentation (Months)	20.5	26.9	28.1	35.0	31.9	49.3	23.5	52.6
Birth Weight (Grams)	1675.0	919.9	2829.3	901.9	2192.4	1089.0	2909.2	946.1
NICU Stay (Days)	60.5	73.2	30.4	32.7	37.0	33.8	46.0	54.7

Mean birth weight differed across subtypes: 1,675 g (SD 919) in SD, 2,829 g (SD 901) in SH, 2,192 g (SD 1,089) in SQ, and 2,909 g (SD 946) in unclassifiable CP.

Mean NICU stay was 43.4 days (SD 51.3). Mode of delivery included vaginal delivery (58.7%), elective lower-segment caesarean sections (LSCS) (40.9%), and emergency lower-segment caesarean sections (ELSCS) (0.4%) (Table 2).

**Table 2:** Clinical characteristics and functional classification by CP subtype (*N* = 179).

Clinical characteristic	Category	Types of cerebral palsy							
		Spastic diplegia *n* = 62		Spastic hemiplegia *n* = 24		Spastic quadriplegia *n* = 72		Unspecified *n* = 21	
		Count	Percentage	Count	Percentage	Count	Percentage	Count	Percentage
Gender	Male	38	61.3%	13	54.2%	47	65.3%	13	61.9%
	Female	24	38.7%	11	45.8%	25	34.7%	8	38.1%
Nationality	Expats	36	58.1%	13	54.2%	51	70.8%	16	76.2%
	UAE	26	41.9%	11	45.8%	21	29.2%	5	23.8%
GMFCS	I	3	11.5%	5	50.0%	0	0.0%	0	0.0%
	II	5	19.2%	4	40.0%	0	0.0%	0	0.0%
	III	6	23.1%	0	0.0%	4	14.8%	0	0.0%
	IV	6	23.1%	1	10.0%	3	11.1%	1	33.3%
	V	5	19.2%	0	0.0%	20	74.1%	2	66.7%
Gestational Age	Extremely Preterm	9	14.5%	0	0.0%	10	13.9%	1	4.8%
	Late Preterm	8	12.9%	3	12.5%	4	5.6%	0	0.0%
	Moderately Preterm	7	11.3%	0	0.0%	4	5.6%	0	0.0%
	Preterm	1	1.6%	0	0.0%	0	0.0%	0	0.0%
	Term	24	38.7%	18	75.0%	45	62.5%	17	81.0%
	Very Preterm	13	21.0%	3	12.5%	9	12.5%	3	14.3%
Type of Delivery	ELSCS	1	1.6%	0	0.0%	0	0.0%	0	0.0%
	LSCS	25	40.3%	10	41.7%	28	38.9%	9	42.9%
	Vaginal	36	58.1%	14	58.3%	44	61.1%	12	57.1%

Values are presented as counts and percentages. Percentages were calculated using the total number of patients within each CP subtype. Percentages for GMFCS are based on available data only.

Antenatal factors were identified in 65 cases (36.3%) (Table 3). Birth-related complications were present in 20 (11.2%), and maternal anaemia or hypertension in 9 (5.1%). Intrauterine infections were noted in 5 cases (2.8%) where congenital cytomegalovirus (*n* = 2), urinary tract infection (*n* = 1), and unspecified (*n* = 2). Maternal autoimmune conditions were implicated in 2 cases (1.1%), and genetic factors in 28 (15.6%).

**Table 3:** Antenatal and maternal risk factors by CP subtype (*N* = 179).

Characteristic	Category	Types of cerebral palsy							
		Spastic diplegia *n* = 62		Spastic hemiplegia *n* = 24		Spastic quadriplegia *n* = 72		Unspecified *n* = 21	
		Count	Percentage	Count	Percentage	Count	Percentage	Count	Percentage
Birth Related Complication	Absent	51	82.3%	23	95.8%	65	90.3%	20	95.2%
	Present	11	17.7%	1	4.2%	7	9.7%	1	4.8%
Maternal Anemia/Hypertension	Absent	56	90.3%	22	95.7%	70	97.2%	21	100.0%
	Present	6	9.7%	1	4.3%	2	2.8%	0	0.0%
Infection	Absent	58	93.5%	24	100.0%	71	98.6%	21	100.0%
	Present	4	6.5%	0	0.0%	1	1.4%	0	0.0%
Maternal Autoimmune Conditions	Absent	60	96.8%	24	100.0%	72	100.0%	21	100.0%
	Present	2	3.2%	0	0.0%	0	0.0%	0	0.0%

Values are presented as counts and percentages. Percentages were calculated using the total number of patients within each CP subtype, and are based on available data; missing data were excluded where applicable.

Perinatal asphyxia was the most common perinatal or neonatal risk factor, affecting 47 children (26.3%) (Table 4). HIE was present in 26 cases (14.5%), neonatal jaundice in 25 (14.0%), and IVH in 17 (9.5%). Perinatal stroke was implicated in 5 cases (2.8%), all within the SH subtype (20.8% of SH), with no cases recorded in SD, SQ, or unspecified CP. Only 2 cases were attributed to trauma or post-natal injury.

**Table 4:** Perinatal and postnatal risk factors by CP subtype (*n* = 179).

Risk factor	Status	Types of cerebral palsy							
		Spastic diplegia *n* = 62		Spastic hemiplegia *n* = 24		Spastic quadriplegia *n* = 72		Unspecified *n* = 21	
		Count	Percentage	Count	Percentage	Count	Percentage	Count	Percentage
Neonatal Jaundice	Absent	51	82.3%	21	87.5%	63	87.5%	19	90.5%
	Present	11	17.7%	3	12.5%	9	12.5%	2	9.5%
Trauma	Absent	61	98.4%	24	100.0%	70	98.6%	21	100.0%
	Present	1	1.6%	0	0.0%	1	1.4%	0	0.0%
Hypoxic Ischemic Encephalopathy	Absent	56	90.3%	23	95.8%	56	77.8%	18	85.7%
	Present	6	9.7%	1	4.2%	16	22.2%	3	14.3%
Intraventricular Hemorrhage	Absent	55	88.7%	20	83.3%	67	93.1%	20	95.2%
	Present	7	11.3%	4	16.7%	5	6.9%	1	4.8%
Perinatal Asphyxia	Absent	49	79.0%	21	87.5%	48	66.7%	14	66.7%
	Present	13	21.0%	3	12.5%	24	33.3%	7	33.3%
Neonatal Infection	Absent	56	90.3%	23	95.8%	65	90.3%	19	90.5%
	Present	6	9.7%	1	4.2%	7	9.7%	2	9.5%
Genetics	Absent	55	88.7%	22	91.7%	60	83.3%	14	66.7%
	Present	7	11.3%	2	8.3%	12	16.7%	7	33.3%
Perinatal Stroke	Absent	62	100.0%	19	79.2%	72	100.0%	21	100.0%
	Present	0	0.0%	5	20.8%	0	0.0%	0	0.0%

Values are presented as counts and percentages. Percentages were calculated within each CP subtype using available data; missing data were excluded where applicable.

### Clinical characteristics

The Gross Motor Function Classification System (GMFCS) level distribution across the full cohort was: level I 12.3%, level II 13.8%, level III 15.4%, level IV 16.9%, and level V 41.5% (Table 2). Among SH children, 90% fell within levels I or II. In SQ and unspecified CP, 74% and 66% respectively were at level V, compared to 20% of SD children. No SQ or unspecified CP child was at level I or II.

### Association with comorbidities

The specifics of the percentages and number of children with each comorbidity are described in Table 5. Differences in the prevalence of several comorbidities were observed across CP subtypes. Epilepsy demonstrated variation across subtypes (p < 0.001), with the highest prevalence observed in SQ. Abnormal gait, feeding difficulties, dysmorphic features, and gastrointestinal abnormalities also showed variation across subtypes (*p* < 0.05 for all), with higher observed prevalence generally noted in SQ compared to other subtypes. In contrast, global developmental delay showed no clear difference across CP subtypes (*p* = 0.192).

**Table 5:** Prevalence of comorbidities by CP subtypes (*N* = 179).

Comorbidity	Status	Types of cerebral palsy								
		Spastic diplegia *n* = 62		Spastic hemiplegia *n* = 24		Spastic quadriplegia *n* = 72		Unspecified *n* = 21		
		Count	Percentage	Count	Percentage	Count	Percentage	Count	Percentage	*P*-value
Global Developmental Delay	Absent	13	21.3%	9	37.5%	12	16.7%	4	19.0%	0.192
	Present	48	78.7%	15	62.5%	60	83.3%	17	81.0%	
Epilepsy/Seizures	Absent	45	73.8%	12	50.0%	23	31.9%	6	30.0%	**<0** **.** **001**
	Present	16	26.2%	12	50.0%	49	68.1%	14	70.0%	
Dystonia	Absent	30	48.4%	13	54.2%	30	41.7%	7	33.3%	0.462
	Present	32	51.6%	11	45.8%	42	58.3%	14	66.7%	
Musculoskeletal Abnormalities	Absent	24	38.7%	15	62.5%	26	36.6%	11	52.4%	0.106
	Present	38	61.3%	9	37.5%	45	63.4%	10	47.6%	
Visual Impairments	Absent	34	54.8%	15	62.5%	40	55.6%	12	57.1%	0.930
	Present	28	45.2%	9	37.5%	32	44.4%	9	42.9%	
Autism	Absent	52	83.9%	22	91.7%	70	97.2%	19	90.5%	0.062
	Present	10	16.1%	2	8.3%	2	2.8%	2	9.5%	
Abnormal Gait	Absent	31	50.0%	5	20.8%	50	69.4%	14	66.7%	**<0** **.** **001**
	Present	31	50.0%	19	79.2%	22	30.6%	7	33.3%	
Urinary Incontinence	Absent	57	91.9%	24	100.0%	58	80.6%	18	85.7%	0.048
	Present	5	8.1%	0	0.0%	14	19.4%	3	14.3%	
Scoliosis	Absent	52	83.9%	20	83.3%	56	77.8%	17	81.0%	0.825
	Present	10	16.1%	4	16.7%	16	22.2%	4	19.0%	
Hip Displacement	Absent	53	85.5%	24	100.0%	61	84.7%	18	85.7%	0.250
	Present	9	14.5%	0	0.0%	11	15.3%	3	14.3%	
Feeding Difficulties	Absent	41	66.1%	16	66.7%	27	37.5%	10	47.6%	**0** **.** **004**
	Present	21	33.9%	8	33.3%	45	62.5%	11	52.4%	
Drooling	Absent	48	77.4%	21	87.5%	54	75.0%	14	66.7%	0.413
	Present	14	22.6%	3	12.5%	18	25.0%	7	33.3%	
Sleeping Difficulties	Absent	51	82.3%	21	87.5%	58	80.6%	18	85.7%	0.859
	Present	11	17.7%	3	12.5%	14	19.4%	3	14.3%	
Respiratory Issues	Absent	47	75.8%	22	91.7%	47	65.3%	15	71.4%	0.081
	Present	15	24.2%	2	8.3%	25	34.7%	6	28.6%	
ADHD	Absent	58	93.5%	23	95.8%	70	97.2%	20	95.2%	0.786
	Present	4	6.5%	1	4.2%	2	2.8%	1	4.8%	
Dysmorphic Features	Absent	58	93.5%	23	95.8%	64	88.9%	13	61.9%	**<0** **.** **001**
	Present	4	6.5%	1	4.2%	8	11.1%	8	38.1%	
GI Abnormalities	Absent	55	88.7%	23	95.8%	44	61.1%	11	52.4%	**<0** **.** **001**
	Present	7	11.3%	1	4.2%	28	38.9%	10	47.6%	
Speech Impairment	Absent	42	67.7%	12	52.2%	38	52.8%	12	57.1%	0.315
	Present	20	32.3%	11	47.8%	34	47.2%	9	42.9%	
Cardiovascular	Absent	60	96.8%	23	95.8%	63	87.5%	19	90.5%	0.210
	Present	2	3.2%	1	4.2%	9	12.5%	2	9.5%	
Hearing Impairments	Absent	59	95.2%	23	95.8%	68	94.4%	19	90.5%	0.855
	Present	3	4.8%	1	4.2%	4	5.6%	2	9.5%	
Coordination	Absent	54	87.1%	16	66.7%	60	83.3%	15	75.0%	0.139
	Present	8	12.9%	8	33.3%	12	16.7%	5	25.0%	

Values are presented as counts and percentages. Percentages were calculated within each CP subtype using available data; missing data were excluded where applicable. *P*-values were calculated using chi-square test as appropriate; these analyses are exploratory and should be interpreted accordingly.

Bold values indicate statistical significance (*p* < 0.05).

#### Global developmental delay (GDD)

GDD was the most common comorbidity, found in 140 of 178 children (78.7%). Rates were highest in SQ (83.3%, 60/72) and unspecified CP (81.0%, 17/21), followed closely by SD (78.7%, 48/61). SH had the lowest prevalence at 62.5% (15/24).

#### Epilepsy

Epilepsy was present in 91 of 178 children (51.1%), of whom 58 of 91 (63.7%) had drug-resistant epilepsy or intractable seizures. Although epilepsy was observed in a substantial portion of our patients, prevalence rates varied across CP subtypes: SQ had the highest prevalence, affecting 49 patients (68.1%), followed by SH at 50% (12 patients), SD at 26.2% (16 patients), and unspecified CP (U) at 70% (14 patients). Subtype-specific data were unavailable for all cases, and therefore subgroup totals do not sum to the overall epilepsy prevalence.

#### Autism and ADHD

Autism was seen in 16 of 179 children (8.9%). SD had the highest rate at 16.1% (10/62), while other subtypes ranged from 2.8% to 9.5%. ADHD was less common still, affecting 8 patients (4.5%); SD again had the highest rate at 6.5% (4/62), and SQ the lowest at 2.8% (2/72).

#### Dystonia

Dystonia was noted in 99 of 179 children (55.3%). Unspecified CP had the highest rate, with two-thirds of children affected (66.7%, 14/21), followed by SQ at 58.3% (42/72), SD at 51.6% (32/62), and SH at 45.8% (11/24).

#### Gait and coordination

Gait abnormalities were reported in 79 of 179 children (44.1%). SH was the most affected subtype (79.2%, 19/24), while in SD half the children had gait issues (50.0%, 31/62). Coordination difficulties were less common, affecting 33 children (18.4%); SH again had the highest rate at 33.3% (8/24).

#### Visual and hearing impairment

Visual impairment affected 78 of 179 children (43.6%) where SD 45.2% (28/62), SQ 44.4% (32/72), unspecified CP 42.9% (9/21), and SH 37.5% (9/24). Among those affected, 22 had refractive errors, and 30 has strabismus. A further 33 children had other visual issues: nystagmus (*n* = 4), refractive errors such as astigmatism or myopia (*n* = 3), optic disc abnormalities including pallor or atrophy (*n* = 3), and central or severe vision loss (*n* = 4). Nineteen patients had miscellaneous conditions including esotropia, retinopathy of prematurity, panuveitis, and ocular prosthesis. Some patients had multiple overlapping visual conditions; therefore, subcategories do not sum to the total. Hearing impairment was identified in 10 children (5.6%): SD 4.8% (3/62), SH 4.2% (1/24), SQ 5.6% (4/72), and unspecified CP 9.5% (2/21).

#### Speech impairment

Speech impairment was one of the major challenges in which it affected 74 of 179 children (41.3%). It was most common in SH at 47.8% (11/23), followed by SQ at 47.2% (34/72), unspecified CP at 42.9% (9/21), and SD at 32.3% (20/62).

#### Hip displacement, scoliosis, and musculoskeletal abnormalities

Musculoskeletal abnormalities were present in 102 of 178 children with available data (57.3%). The highest prevalence was observed in SQ (45/71, 63.4%), followed by SD (38/62, 61.3%), unspecified CP (10/21, 47.6%), and SH (9/24, 37.5%). Distinctly, scoliosis was documented in 34 of 179 children (19.0%), and hip displacement in 23 of 179 children (12.8%).

#### Feeding and drooling

Feeding difficulties and drooling were reported in 85 of 179 children (47.5%). SQ was most affected at 43.1% (31/72), followed by SD at 25.8% (16/62), and SH at 12.5% (3/24). Unspecified CP had 23.8% (5/21).

#### Respiratory and cardiovascular issues

Respiratory and cardiovascular complications were recorded in 48 of 179 children (26.8%). SQ had the highest rate at 34.7% (25/72), followed by SD at 16.1% (10/62), and SH at 8.3% (2/24). In Unspecified CP, respiratory or cardiovascular complications were seen in 28.6% (6/21) of children.

#### GI abnormalities and urinary incontinence (visceral dysfunction)

Visceral dysfunction encompassing GI abnormalities and urinary incontinence was found in 46 of 179 children (25.7%). SQ had the highest prevalence at 33.3% (24/72), followed by SD at 16.1% (10/62), and SH at 8.3% (2/24). Unspecified CP presented with a prevalence of 23.8% (5/21).

#### Sleeping difficulties

Sleep difficulties were reported in 31 of 179 children (15.1%). SQ was most affected, with 23.6% (17/72) of children reporting disturbed sleep, likely related to discomfort and spasticity. SD had a rate of 11.3% (7/62), and SH the lowest at 4.2% (1/24). The group of unspecified CP demonstrated a prevalence of 14.3% (3/21).

### Rehabilitation programs

Of the 179 children, 116 (64.8%) received physiotherapy (PT), the most commonly accessed service. Occupational therapy (OT) was attended by 66 children (36.9%), speech-language therapy (SLT) by 53 (29.6%), and feeding therapy (FT) by 28 (15.6%).

## Discussion

### Summary of major findings

179 children with a confirmed diagnosis of CP were assessed at a tertiary care hospital in the UAE to evaluate associated comorbidities, functional limitations, and the use of multiple rehabilitation services. The most common subtypes were SQ, SD, and SH. The main documented etiological factors included perinatal asphyxia, neonatal jaundice, and HIE, some of which may be influenced by antenatal and perinatal care.

Common comorbidities included GDD, epilepsy, visual impairment, and gait abnormalities. Regarding the rehabilitation facilities availed, 64.8% of the cohort participated in physiotherapy, reflecting moderate participation and suggesting potential gaps in access to supportive care and resource distribution.

Several comorbidities revealed observed variation by CP subtype, particularly in SQ. These findings show the burden of comorbidities among children with CP and underscore the importance of continued efforts in primary prevention, comprehensive care, and equitable access to rehabilitation services.

### Comparison with previous studies

The current study findings align with the global literature, particularly the hospital-based study by Viswanath et al. from India, which reported a high prevalence of severe CP subtypes such as SQ and SD, along with frequent co-occurrence of associated perinatal factors such as perinatal asphyxia and neonatal hypoglycemia (14). Perinatal asphyxia accounted for 26.3% of cases in our cohort, similar to the 23.4% reported in the Indian study, suggesting that perinatal hypoxic events may represent an important associated factor across different healthcare settings. On the other hand, 8.9% of the current cohort had neonatal infections, which was lower compared to the 19.3% reported in India; these differences may reflect variations in regional antibiotic resistance patterns or, in fact, the overall severity of the cases (14).

In Sweden, Himmelmann et al. reported GDD in 45% of children with CP, while in the current study cohort, it was higher, with 80% prevalence. This may reflect varying levels of access to care, case severity, and regional differences between hospital-based versus population-based studies in CP aetiology. Furthermore, Himmelmann et al. reported that a majority, 58%, of their cohort had Gross Motor Function Classification System (GMFCS) levels of 4 or higher, indicating significant functional impairment. Our study revealed that 62.2% of the children exhibited similar functional impairments, with SQ and unspecified CP subtypes being associated with greater functional difficulties. This leads to the same trend as in the Swedish study, which also aligns with more prior research studies, supporting previously reported patterns of variation in functional outcomes across CP subtypes (19, 20).

Access to rehabilitation was higher than in LMICs such as India and Bangladesh but remained below levels reported in high-income settings (14). On the contrary, Himmelmann et al. cited higher percentages of multidisciplinary interventions in Sweden, suggesting that robust healthcare systems can mitigate some of the burdens associated with CP in high-income countries. Such disparities raise the need to increase resource allocation and access to health services in developing and middle-income nations (19, 21).

In Bangladesh, 78% of children with cerebral palsy had never received any form of rehabilitation, largely due to a lack of awareness, financial restraints, and barriers like difficult transportation (22). These findings accentuate the many challenges of addressing the comprehensive needs of children with CP, with socioeconomic factors and resource allocation playing major roles in shaping access to care.

These findings emphasize the importance of addressing potentially preventable perinatal risk factors and improving access to multidisciplinary care.

The higher prevalence of multiple comorbidities in SQ highlights the need for early assessment and targeted management strategies in this subgroup. Thus, these findings emphasize the value of regular screening for such co-existing conditions, alongside personalized treatment plans that include rehabilitation programs tailored to the diverse needs of patients with CP.

### Strengths and limitations

This study offers valuable insights into the prevalence and characteristics of CP in the UAE, using data from a diverse cohort to characterize comorbidities, aetiologies, and varying limitations in activities of daily living. The hospital-based setting enabled detailed analysis and sufficient data collection, contributing to the study's strengths.

This hospital-based, single-center study has intrinsic limitations, including referral bias that likely overrepresents severe cases while underrepresenting milder forms of CP and individuals who did not seek care (participation bias). The retrospective nature of data collection limits the ability to establish causal relationships. Furthermore, the observed burden of comorbidities is likely confounded by overall disease severity (e.g., GMFCS level), gestational age, and prematurity. Because sample size constraints within individual CP subtypes limited our ability to perform robust multivariable logistic regression, our findings regarding subtype-specific comorbidity rates must be viewed as exploratory associations rather than isolated effects of the subtype itself. Misclassification bias may also be present. Additionally, multiple statistical comparisons were performed, and certain statistical assumptions (such as adequate cell counts) were not met due to the cohort size. This increases the risk of Type 1 errors; therefore, the findings should be interpreted exclusively as exploratory and descriptive. Consequently, conclusions regarding preventive policies and access disparities are observational and cannot establish definitive population prevalence or causation.

Moreover, the single-center design means that the findings may not be representative of the entire population and are not generalizable to community settings or regions with different healthcare systems. However, the hospital-based design also provides an advantage by enabling focused analysis of children with more severe disabilities. It may also help clinicians identify coexisting conditions and optimize the use of healthcare resources. Moreover, the documented etiological factors may help inform future quality-improvement efforts and identify potential gaps in early neonatal care (14).

Future research should examine large-scale population-based samples to better assess the generalizability of the findings on the true incidence and severity of CP. Future research should address several areas, including the need for long-term observational studies of comorbidities and the efficacy of early interventions, such as physiotherapy and epilepsy care. Population-based CP genetic research focusing on the possible role of heritability in CP in the UAE may provide further insight into regional etiological patterns and inform future preventive strategies.

Studies should be conducted to understand the socioeconomic and cultural factors that deny people access to rehabilitation interventions, with the aim of informing policy on justice in the provision of rehabilitation services. Additionally, investigations into caregiver stress and support systems may provide valuable insights to enhance family-centered approaches to care. Lastly, conducting a study on the transition of children with CP from paediatric to adult healthcare would thus fill gaps regarding service delivery and comprehensive lifelong care.

## Conclusions

The findings of the present research describe the high observed prevalence of comorbidities in children with CP in this tertiary-care cohort in the UAE, primarily associated with severe forms of CP and specific perinatal factors. In conclusion, children with CP can have co-occurring impairments (neurological, psychological, behavioral, and medical) for which they require detailed comprehensive management measures.

The etiology of CP is extremely complex with major scientific advancements constantly in play to help in understanding these factors, thus continued efforts are needed to address associated disabilities that further complicate the clinical picture. The results share similarities with findings from other LMICs; however, comorbidities and rehabilitation opportunities may vary across regions, highlighting the specific issues of this population. Multisectoral public health approaches involving healthcare providers, educators, and policymakers may help address these challenges.

In terms of implications for future practice, this study provides preliminary regional data to identify key areas for intervention and to highlight the significance of holistic, family-centered approaches to enhancing the quality of life for children with CP and their families. The present study provides directions for future research to address the remaining gaps in the evidence base and to develop tailored strategies for effective CP prevention and management.

## Data Availability

The raw data supporting the conclusions of this article will be made available by the authors, without undue reservation.
