# Supplementary material for: Beyond motor dysfunction: comorbidities in children with cerebral palsy: a retrospective observational study from a tertiary paediatric hospital in the UAE
Source: Front Pediatr. 2026 Jul 22;14:1776355. doi: 10.3389/fped.2026.1776355 (PMC13437961; doi:10.3389/fped.2026.1776355)
Supplement: Supplementary file 1 [file Table1.docx]

Supplementary Table S1: Operational Definitions of Pertinent Comorbidities

| Comorbidities | Operational Definition |
| --- | --- |
| Global Developmental Delay | Documented significant delay in ≥2 developmental domains for children <5 years of age; for children ≥5 years, documented intellectual disability or persistent global developmental impairment was used where formal IQ testing was unavailable. The term ‘GDD’ was used in the dataset for consistency, acknowledging that formal IQ testing was not consistently documented [23]. |
| Epilepsy/Seizures | Documented epilepsy by the pediatric neurology department and/or recurrent unprovoked seizures consistent with the International League Against Epilepsy’s (ILAE) practical clinical definition [24]. |
| Dystonia | Documented dystonia on neurological exam, consistent with the widely accepted consensus definition: persistent or intermittent muscle contractions resulting in abnormal, repetitive movements or postures [25]. |
| Visual Impairments | Documented visual impairment based on clinical diagnosis or ophthalmologic assessment; this was defined, according to the WHO, as best-corrected visual acuity < 6/18 in the better eye and/or the presence of clinically significant visual dysfunction, including cerebral visual impairment, strabismus, or visual field defects [26]. Refractive errors alone were not classified as visual impairment unless visual acuity remained reduced after correction. |
| Autism | Documented diagnosis of autism spectrum disorder by a qualified clinician, consistent with DSM-5 framework [27]. |
| Abnormal Gait | Documented abnormal gait pattern (e.g., spastic, ataxic, crouch gait) noted in clinical or physiotherapy assessments. |
| Urinary Incontinence | Documented involuntary loss of urine inappropriate for age and developmental status. |
| Scoliosis | Documented scoliosis diagnosis; according to radiology notes, scoliosis was commonly defined as Cobb angle ≥10°, and this was specified for some, not all patients, and the latter was mainly clinical diagnosis. |
| Hip Displacement | Documented hip subluxation or dislocation confirmed by orthopedic or radiologic evaluation; specific indices (migration percentage) were not consistently available. |
| Feeding Difficulties | Documented clinically significant oromotor dysfunction requiring intervention, including tube feeding (NG/PEG), documented aspiration risk, or feeding assessed as unsafe/inefficient by a clinician |
| Drooling | Documented persistent drooling in clinical notes which in most cases required medical/therapeutic intervention. |
| Sleeping Difficulties | Documented sleep disturbances including insomnia, frequent awakenings, or sleep-disordered breathing. |
| Respiratory Issues | Documented presence of physician-diagnosed chronic respiratory disease such as asthma, recurrent lower respiratory tract infections (≥2/year requiring treatment), aspiration-related respiratory disease, or need for long-term respiratory support [28]. |
| ADHD | Documented diagnosis of ADHD by a qualified clinician/team using the AAP or DSM-5 diagnostic criteria. |
| Dysmorphic Features | Documented presence of congenital or syndromic physical features noted on clinical examination. |
| GI Abnormalities | Documented physician-diagnosed chronic constipation requiring treatment, gastroesophageal reflux disease requiring medication, or feeding-related aspiration/GERD complications. |
| Speech Impairment | Documented impairment in expressive or receptive language skills based on clinical or speech therapy assessment. |
| Cardiovascular | Documented structural or functional cardiac abnormalities diagnosed by a pediatric cardiologist. |
| Hearing Impairments | Documented hearing loss confirmed by audiological testing or clinician diagnosis, where applicable based on patient age and type of CP. |
| Coordination | Documented impairment in coordination or motor planning based on neurological or physiotherapy assessment. |
